# Caffeine-Induced Severe Erosive Esophagitis

**DOI:** 10.7759/cureus.16253

**Published:** 2021-07-08

**Authors:** Justice S Arhinful, Benedicta Arhinful, Timi A Akins, Sarah Hossain

**Affiliations:** 1 Internal Medicine, University of Missouri School of Medicine, Springfield Clinical Campus, Springfield, USA; 2 Hospital Medicine/Internal Medicine, Lester E. Cox Medical Center South, Springfield, USA; 3 Health Policy and Management, Johns Hopkins Bloomberg School of Public Health, Baltimore, USA; 4 Nephrology, Ferrell-Duncan Clinic, Springfield, USA; 5 Gastroenterology, Ferrell-Duncan Clinic, Springfield, USA

**Keywords:** esophagitis, caffeine, overdose, esophageal injury, gastrointestinal

## Abstract

Caffeine is one of the most frequently used stimulants worldwide. It is, therefore, subject to frequent intentional and unintentional misuse. However, severe erosive esophagitis due to acute caffeine overdose is extremely rare. We report the case of a 43-year-old male with a past medical history of paranoid schizophrenia admitted to our hospital with esophageal symptoms (throat pain, retrosternal chest pain, dysphagia/odynophagia, nausea, and vomiting) two days after ingesting a bottle of caffeine pills containing about 30 g of caffeine in a suicide attempt. He was found to have rhabdomyolysis and acute renal failure warranting hemodialysis. Esophagogastroduodenoscopy done due to persistent retrosternal chest pain, dysphagia, odynophagia, and nausea despite being on oral famotidine 20 mg daily revealed severe erosive esophagitis. This case highlights the risk of concurrent renal and gastrointestinal injuries after acute ingestion of an excessive amount of caffeine tablets. Our experience suggests that in patients of caffeine overdose with persistent esophageal symptoms such as odynophagia, dysphagia, and retrosternal chest pain, endoscopic evaluation is advisable to rule out drug-induced esophagitis.

## Introduction

Caffeine (1,3,7-trimethylxanthine) is a ubiquitous natural xanthine alkaloid found in coffee, cocoa, tea, and kola nuts [1-3]. It is one of the most widely utilized stimulants worldwide for several years due to its capacity to improve mental alertness [1-4]. Approximately 85% of Americans consume caffeine daily with about 40-150 mg of caffeine in an average cup of coffee [4]. Because caffeine is found in several substances (including prescription and over-the-counter drugs, energy drinks, appetite suppressants, and food, and weight loss supplements), the potential of toxicity with an unintentional overuse or severe toxicity with intentional overdose is very high [4]. Although caffeine is generally considered to be safe in small-to-moderate amounts, caffeine overdose may result in varied clinical features, including gastrointestinal, neurologic, or cardiovascular symptoms [3,5]. While caffeine overdose frequently induces gastrointestinal symptoms, such as nausea, vomiting, and epigastric pain, the number of patients who experience significant esophageal injuries is extremely low [6]. Thus, the pathophysiologic and endoscopic features of caffeine-induced esophageal injury remain unclear. We report a case of severe erosive esophagitis caused by intentional ingestion of excessive caffeine tablets.

## Case presentation

A 43-year-old male with a past medical history of paranoid schizophrenia presented to the emergency department (ED) with esophageal symptoms (throat pain, dysphagia/odynophagia, nausea, and vomiting) two days after ingesting a bottle of caffeine pills (about 30 g of caffeine) in an apparent suicide attempt. His vital signs on presentation to the ED included a blood pressure of 106/70 mmHg, heart rate of 136 beats per minute, body temperature of 98.5°F, and saturation of 91% on room air. His abdomen was soft and non-distended but was mildly tender to palpation in the epigastric region with no rebound or guarding; his bowel sounds were normal. There were no respiratory or cardiovascular findings. His Glasgow Coma Scale was 15. All other physical examination findings were normal.

Pertinent laboratory findings were white blood cell count of 11.9 × 10^9^/L (neutrophils 89.9%, lymphocytes 3.7%, monocytes 5.7%), hemoglobin 20.0 g/L, platelet 241 × 10^9^/L, sodium 134 mEq/L, potassium 3.7 mEq/L, chloride 86 mEq/L, bicarbonate 40 mEq/L, blood urea nitrogen 44 mg/dL, creatinine 3.9 mg/dL, aspartate aminotransferase 83 U/L, alanine aminotransferase 44 U/L, and creatinine kinase of 2,588 U/L. The urine drug screen was negative. Urine chemistry was suggestive of acute tubular necrosis. Urinalysis showed 2+ proteinuria and trace ketonuria, but was otherwise unremarkable. Electrocardiography showed a normal sinus rhythm. The patient’s blood caffeine level was not assessed.

He was found to have rhabdomyolysis and acute renal failure. Despite receiving aggressive intravenous fluid hydration in the ED with 2 L of normal saline intravenous (IV) bolus followed by lactated ringers at 125 cc/hour on the medical floor, his renal function continued to worsen peaking at 7.61 mg/dL on day three of the admission, warranting initiation of hemodialysis. His renal failure gradually recovered after three months of intermittent hemodialysis. A renal biopsy was not performed.

**Figure 1:**
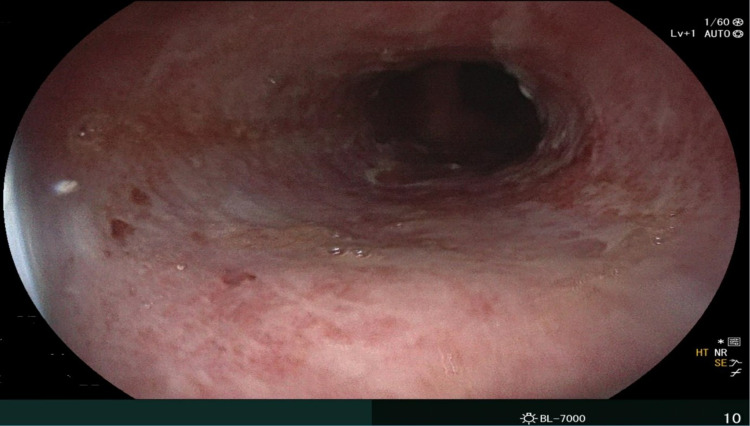
The esophagus showing circumferential erosive changes from 29 to 28 cm, with ulceration in the distal third of the esophagus suggestive of severe erosive esophagitis.

Esophagogastroduodenoscopy (EGD) done on day seven of hospital stay due to persistent esophageal symptoms despite being on famotidine showed severe erosive esophagitis (Figure 1) and gastric subepithelial lesion​. Pathology reports of the gastric biopsy were consistent with mild chronic inactive gastritis, while that of the gastric subepithelial lesion was consistent with leiomyoma. His esophageal symptoms markedly improved following the initiation of proton pump inhibitor (pantoprazole 40 mg IV twice daily). He did not show up for a repeat EGD. This case highlights the risk of concurrent renal and gastrointestinal injuries after ingesting excessive caffeine tablets. Our experience suggests that for patients who have ingested excessive caffeine tablets, endoscopic evaluation is advisable in the setting of persistent esophageal symptoms to evaluate for chemical esophagitis.

## Discussion

Caffeine is a stimulant that has been utilized worldwide for several centuries [1-3]. Owing to the widespread availability of caffeine in various forms, intentional or intentional caffeine overdose is more than frequent [4]. Here, we have presented a very rare case of severe erosive esophagitis due to caffeine overdose. To the best of our knowledge, only one case of esophageal injury secondary to an overdose of caffeine tablets has been reported in the English literature; three other cases of esophageal injury secondary to caffeine overdose have been reported in Japanese [6]. Miyata et al. reported the case of a 19-year-old female who developed diffuse lower esophageal ulcers without any gastric ulcers or erosions following the consumption of 24 tablets of Estaron-mocha (SSP Co. Ltd., Chuo-Ku, Japan) amounting to 2.4 g of caffeine in an apparent suicide attempt [6].

Pill-induced esophagitis is an esophageal mucosal injury that is usually caused by a direct toxic effect on the esophageal mucosa by the culprit medications [7]. Some of the risk factors associated with pill-induced esophagitis include esophageal dysmotility, large pills, decreased salivary output, medications that increase the lower esophageal sphincter tone, and ingestion of medications in the supine position [7]. Symptoms commonly reported by patients include nausea, vomiting, retrosternal chest pain, odynophagia, and dysphagia occurring several hours to days after the ingestion of medication. Some of the medications known to cause pill-induced esophagitis include bisphosphonates, quinidine, and mexiletine. Many of the medications most frequently implicated to cause pill-induced esophagitis have deleterious effects on the esophageal mucosa, including the production of local hyperosmolarity (as seen with potassium chloride) and alteration of pH and osmolarity (as in the case of ferrous sulfate), whereas nonsteroidal anti-inflammatory drugs and doxycycline induce esophageal injury via intracellular uptake and cytotoxicity [7]. However, the mechanisms of mucosal injury caused by some other medications, for example, caffeine pills, are unclear. Caffeine is known to stimulate gastrin release and gastric acid secretion which may play a role in its toxic effects on the gastrointestinal (GI) tract [3]. Additionally, theophylline, a metabolite of caffeine, is known to induce gastroesophageal reflux when it is dissolved in the GI tract. Because both caffeine and theophylline are structurally and toxicologically similar, caffeine likely shares a similar mechanism of esophageal injury to theophylline [3,6]. Patient-related factors of pill-induced esophagitis, such as ingestion of pills with an inadequate volume of water, may also play a role in the GI toxicity of caffeine [7]. It is likely that patients with suicidal ideations, like our patient, may not have consumed enough water with the ingested caffeine tablets leading to slow esophageal transit time, hence the resultant mucosal injury to the esophagus.

Endoscopy is the gold standard for the diagnosis and management of pill-induced esophagitis [7]. The most common endoscopic findings in patients with pill-induced esophagitis in order of decreasing frequency are esophageal mucosal erythema and erosion, ulcer with or without bleeding, coating with drug material, impacted pill fragments, stricture, and kissing ulcers [7]. These lesions are usually found in the mid-to-lower esophagus [6,7].

In our case, the patient’s esophageal symptoms remained persistent despite being on famotidine. Subsequent diagnostic upper endoscopy revealed severe erosive esophagitis, similar to the common endoscopic findings in patients with caffeine-induced esophagitis. His symptoms improved with a proton pump inhibitor.

## Conclusions

We have described a case of severe erosive esophagitis following acute ingestion of excessive caffeine tablets. This case highlights the risks of esophageal injuries after caffeine overdose, which is readily available. Our experience suggests that endoscopic evaluation is recommended to prevent severe complications if a patient presents with esophageal symptoms concerning for pill-induced esophagitis.
